# Water-dispersible PEG-curcumin/amine-functionalized covalent organic framework nanocomposites as smart carriers for in vivo drug delivery

**DOI:** 10.1038/s41467-018-04910-5

**Published:** 2018-07-17

**Authors:** Guiyang Zhang, Xinle Li, Qiaobo Liao, Yanfeng Liu, Kai Xi, Wenyu Huang, Xudong Jia

**Affiliations:** 1 School of Chemistry and Chemical Engineering, Nanjing University, Jiangsu, 210023 China; 2 Department of Chemistry, Iowa State University, Ames, IA 50011 USA

## Abstract

Covalent organic frameworks (COFs) as drug-delivery carriers have been mostly evaluated in vitro due to the lack of COFs nanocarriers that are suitable for in vivo studies. Here we develop a series of water-dispersible polymer-COF nanocomposites through the assembly of polyethylene-glycol-modified monofunctional curcumin derivatives (PEG-CCM) and amine-functionalized COFs (APTES-COF-1) for in vitro and in vivo drug delivery. The real-time fluorescence response shows efficient tracking of the COF-based materials upon cellular uptake and anticancer drug (doxorubicin (DOX)) release. Notably, in vitro and in vivo studies demonstrate that PEG-CCM@APTES-COF-1 is a smart carrier for drug delivery with superior stability, intrinsic biodegradability, high DOX loading capacity, strong and stable fluorescence, prolonged circulation time and improved drug accumulation in tumors. More intriguingly, PEG_350_-CCM@APTES-COF-1 presents an effective targeting strategy for brain research. We envisage that PEG-CCM@APTES-COF-1 nanocomposites represent a great promise toward the development of a multifunctional platform for cancer-targeted in vivo drug delivery.

## Abstract (web summary)

Despite their potential application as drug-delivery carriers, covalent organic frameworks (COF) have been only evaluated in vitro. Here the authors show by real time tracking in vivo the cell uptake of anticancer-drug loaded and water dispersible COFs.

## Introduction

Covalent organic frameworks (COFs) have garnered considerable interest due to their widespread applications in gas storage^1–3^, optoelectronic and electrical energy storage devices^4–6^, nanochannels^7,8^, catalysis^9–14^, temperature sensing^15^, and photovoltaics^16^. Considering that COFs are solely constructed with organic building blocks^17–21^, exploring their potential in the biomedical field is obvious due to their inherent advantages, such as large surface area, enormous porosity, biocompatibility, and tunable functionality. Recent studies have demonstrated that COFs can serve as drug-delivery carriers with high loading capacity and efficient drug-release behavior^22,23^. However, the intrinsic limitations of COF materials, including their poor physiological stability, nonspecific targeting, and low cell-membrane permeability, severely restrain their practical implementation, and research on COFs in drug delivery remains in its infancy. Despite pioneering studies uncovering the great potential of COFs in drug delivery^22–25^, further optimization in the following aspects must be considered for the design of optimal drug-delivery carriers for in vivo applications. (1) Optimal size: the optimal particle size for efficient cellular uptake is below 200 nm. However, the previously reported COF drug carriers possessed much larger sizes and poor dispersion. (2) Drug loading: plenty of reported loading methods have been based on equilibrium adsorption and diffusion^26,27^, which results in inconsistencies. (3) Good biocompatibility: cytotoxicity in biological systems must be minimized. (4) Controlled drug release: considering the limited number of release strategies employed in COFs materials, realizing a high targeting specificity and controlled drug release remains a challenge but is highly desirable.

On the other hand, owing to the tunable properties of polymers, such as softness, thermal and chemical stability, and photoelectric properties, they have been widely integrated with porous materials to afford nanocomposites with desired properties^28^. Using polymers to modify COFs could be a promising method to adjust the stability, dispersity, and flexibility of these porous crystalline solids. The integration of polymers and porous materials has been reported in metal-organic frameworks (MOFs)/polymer composites^29–31^, which could be pre-prepared^29,30^ or post-prepared^31^ by the formation of MOF crystals using polymeric linkers or the attachment of polymer chains onto the functional ligand of MOF crystals. An amine-functionalized COF-1 has been reported by mixing 3-aminopropyl triethoxysilane (APTES) into established COF-1 recipe via Brønsted-type interactions^25^. In addition, monofunctional curcumin (CCM) derivatives modified by polyethylene glycol (PEG) hold three advantages: (a) the presence of free phenolic groups endows CCM with antioxidant biological activity^32^; (b) CCM could be used as a fluorescent label for tracking; and (c) using monofunctional derivatives to modify polymers could form soluble conjugates in high yields, while using bifunctional derivatives would result in cross-linked products with poor solubility^33^. Taking these factors into consideration, we anticipate that the COF-polymer hybridization could open up a new route for the design of multifunctional nanocomposites for efficient and controllable drug delivery.

**Fig. 1:**
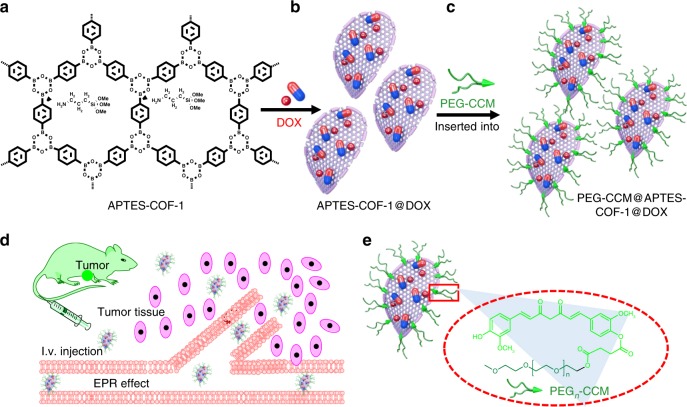
Schematic representations. **a–d** Synthesis of DOX-loaded PEG-CCM@APTES-COF-1 with cellular uptake at weak acidic tumor tissue/cells (i.v. injection intravenous injection). **e** Synthesis of PEG-CCM amphiphilic block copolymers containing hydrophobic blocks based on the unplugging of the PEG-CCM polymer from the APTES-COF-1 pores

In this work, we develop a facile synthesis of a polymer-COF nanocomposite (denoted as PEG-CCM@APTES-COF-1) via the self-assembly of polyethylene-glycol-modified monofunctional curcumin derivatives (PEG-CCM) and amine-functionalized COF‑1 (APTES-COF-1). The entire structure of nanocomposites can be regarded as a micelle with APTES-COF-1 as the oil phase and PEG-CCM as the surfactant (Supplementary Figs 1 and 2). Inside the cell, PEG-CCM is unplugged, and doxorubicin (DOX) is released. Importantly, PEG-CCM@APTES-COF-1 shows strong fluorescence in both the solid state and solution, and the real-time fluorescence response is sufficient for tracking COF-based materials upon cellular uptake and DOX release. Fluorescence tracking experiments indicate that PEG-CCM@APTES-COF-1 efficiently accumulates in tumors after intravenous injection (Fig. 1). Meanwhile, using in vivo experiments, an enhanced tumor-inhibition effect is observed for the DOX-loaded PEG-CCM@APTES-COF-1 nanocarriers compared to the free DOX formulation. Thus, our study presents a facile approach for the synthesis of water-dispersible PEG-CCM@APTES-COF-1 nanocomposites as smart carriers for drug delivery with remarkable anticancer therapeutic efficiency. To the best of our knowledge, this represents a rare example that introduces the emerging research of polymer-COF assembly for therapeutic applications.

## Results

### Characterization of the PEG-CCM@APTES-COF-1 nanocarriers

The synthetic protocol of the PEG-CCM@APTES-COF-1 nanocomposites (denoted as CCM-COOH, PEG_350_-CCM, PEG_1000_-CCM, PEG_2000_-CCM, PEG_350_-CCM@APTES-COF-1, PEG_1000_-CCM@APTES-COF-1, and PEG_2000_-CCM@APTES-COF-1) is illustrated in Supplementary Fig. 1. The ^1^H nuclear magnetic resonance (NMR) spectra of the CCM-COOH, PEG_350_-CCM, PEG_1000_-CCM, and PEG_2000_-CCM derivatives (Supplementary Figs 3 and 4) showed three characteristic signals of CCM at 7.6, 6.5–7.2, and 5.35 ppm, assigned to the –CH=C (2H), Ar (6H), –OH (1H) protons, respectively. In addition, the series of PEG-CCM composites were characterized by electrospray ionization mass spectrometry (ESI-MS) and matrix-assisted laser desorption ionization time-of-flight mass spectrometry (MALDI-TOF MS). From the ESI-MS spectra (Supplementary Figs 5 and 6), the molecular weight evolution from PEG_350_-CCM to PEG_1000_-CCM is clearly observed. However, ESI-MS cannot precisely determine the molecular weight for samples with high molecular weights (>2000). Thus, a more accurate molecular weight of PEG_2000_-CCM was obtained by negative-mode MALDI-TOF MS (Supplementary Fig. 7), confirming that the product is mainly hydrophilic. PEG_2000_-CCM has a long PEG segment and a shorter CCM segment and thus is water soluble.

**Fig. 2:**
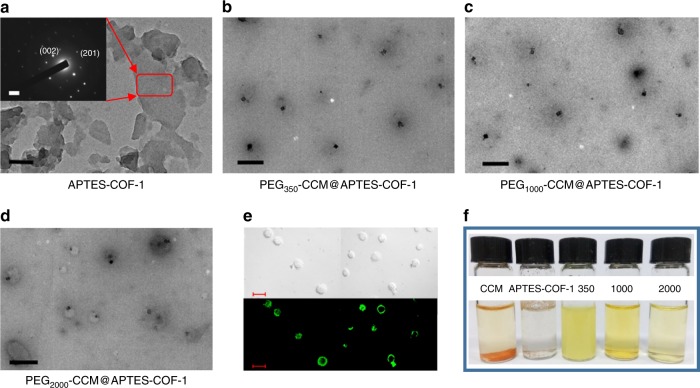
Structural characterizations and digital photo representation. HRTEM images of **a** APTES-COF-1 (scale bar: 100 nm), the inset shows the SAED pattern (scale bar: 0.5 nm). **b** PEG_350_-CCM@APTES-COF-1, **c** PEG_1000_-CCM@APTES-COF-1, and **d** PEG_2000_-CCM@APTES-COF-1. Scale bar: 500 nm. **e** Representative green channel CLSM (Leica TCS SP8 STED 3X, Germany) image recorded for PEG_2000_-CCM@APTES-COF-1 dispersion, the green channel was excited at 488 nm and collected between 510 and 550 nm (scale bar: 200 nm). **f** Digital photographs of dispersions of free CCM, COF-1, PEG_350_-CCM@APTES-COF-1, PEG_1000_-CCM@APTES-COF-1, and PEG_2000_-CCM@APTES-COF-1 in water stored at room temperature for 2 days

The high-resolution transmission electron microscopy (HRTEM) image in Fig. 2a shows that there are many thin platelets with widths ranging from 120 to 150 nm, where the selected area electron diffraction (SAED) pattern indicates its retained crystallinity. To further functionalize the APTES-COF-1, a series of PEG polymers with various molecular weights were first introduced into the APTES-COF-1 vector via self-assembly. In addition to polymer encapsulation, surface modification of COFs with polymers is a fascinating approach to afford COF/polymer composites and imparts COFs with improved properties, such as hydrolytic stability and biocompatibility. Using this approach, a series of samples were prepared via the self-assembly of three PEG-modified CCM derivatives and APTES-COF-1. The monocarboxylic acid derivative of CCM was modified with a pendant carboxylic acid via standard coupling chemistry (Supplementary Fig. 1)^34^. To circumvent the intrinsic obstacles (e.g., low solubility^35^, rapid degradation^34^, low residence time, and fast release kinetics^35–37^), associated with CCM and improve its in vivo therapeutic efficacy, we utilized a covalent conjugation strategy to incorporate biodegradable linkages and thus obtained a series of isoreticular nanocomposites with variable surface PEG densities (PEG_350_-CCM@APTES-COF-1, PEG_1000_-CCM@APTES-COF-1, and PEG_2000_-CCM@APTES-COF-1). Transmission electron microscopy (TEM) images of these PEG-CCM@APTES-COF-1 thin platelets showed monodisperse particles (Fig. 2b–d) with a consistent size of 150 (±8), 170 (±7), and 230 (±10) nm for PEG_350_-CCM@APTES-COF-1, PEG_1000_-CCM@APTES-COF-1, and PEG_2000_-CCM@APTES-COF-1, respectively. The confocal laser scanning microscopy (CLSM) images (Fig. 2e) further showed that PEG_2000_-CCM@APTES-COF-1 particles had a core–shell morphology indicated by the green channel fluorescence. While, without polymer modification, bare APTES-COF-1 was unstable under aqueous physiological conditions, which led to a premature release of drugs or the formation of large aggregates (Fig. 2a) that would impair cellular uptake. Notably, the PEG modification is beneficial to the dispersion of APTES-COF-1 in aqueous media. As shown in Fig. 2f, APTES-COF-1 cannot be dispersed in water, while the PEG-CCM@APTES-COF-1 hybrid exhibited good and stable dispersion in water at room temperature even after a few days. Moreover, the different hydrodynamic sizes of the PEG-CCM@APTES-COF-1 nanoparticles dispersed in aqueous solutions were also measured by dynamic light scattering (DLS) (Supplementary Fig. 8). The hydrodynamic sizes of the PEG-CCM@APTES-COF-1 nanocomposites under pH 7.4 considerably changed with the increasing molecular weight of PEG, which is in accordance with the average particle sizes from the TEM analyses (Fig. 2b–d). All these particles were highly dispersed and stable in water and phosphate-buffered saline (PBS) buffer (pH 7.4), as a result of their *ζ* potentials (+3.1, +3.5, and +3.7 mV for 350, 1000, and 200 assemblies, respectively). As a control, only PEG-CCM spherical micelles were obtained without the addition of APTES-COF-1 into the reaction system (Supplementary Fig. 9). Furthermore, DLS date (Supplementary Fig. 10) of these PEG-CCM spherical micelles showed monodisperse particles with a consistent size of 20 (±5), 30 (±7), and 45 (±10) nm for PEG_350_-CCM, PEG_1000_-CCM, and PEG_2000_-CCM, respectively.

**Fig. 3:**
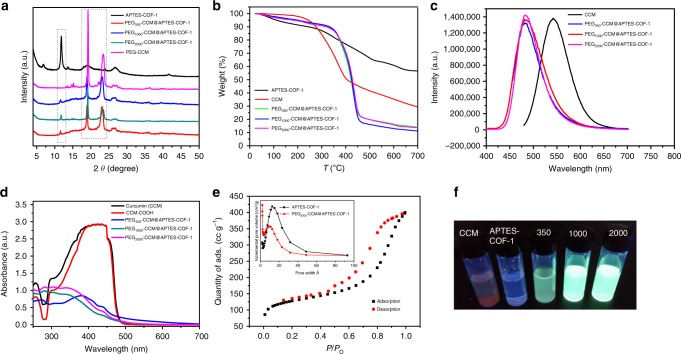
Structural characterizations and fluorescence photograph. **a** PXRD patterns of APTES-COF-1 (black solid line), PEG_350_-CCM@APTES-COF-1, PEG_1000_-CCM@APTES-COF-1, and PEG_2000_-CCM@APTES-COF-1. **b** TGA curves of the as-synthesized CCM, APTES-COF-1, PEG_350_-CCM@APTES-COF-1, PEG_1000_-CCM@APTES-COF-1, and PEG_2000_-CCM@APTES-COF-1 under air atmosphere. **c** PL spectra of free CCM (black solid line), PEG_350_-CCM@APTES-COF-1, PEG_1000_-CCM@APTES-COF-1, and PEG_2000_-CCM@APTES-COF-1. **d** UV−vis spectra of the COF-based materials in methanol solution. **e** N_2_ adsorption (black) and desorption (red) analysis of APTES-COF-1. The inset shows the DFT pore size distribution. **f** Photographs of free CCM, APTES-COF-1, PEG_350_-CCM@APTES-COF-1, PEG_1000_-CCM@APTES-COF-1, and PEG_2000_-CCM@APTES-COF-1 at the same concentration under blue-light excitation (365 nm)

The powder X-ray diffraction (PXRD) pattern of APTES COF-1 showed three major peaks at 11.6°, 19.2°, and 27.5°, corresponding to the 110, 201, and 002 diffraction plane (Fig. 3a). The SAED pattern revealed a fine crystalline structure of APTES-COF-1 in the selected area, within which the electron diffraction signal of 201 and 002 lattice plane can be directly observed, as indicated in the inset of Fig. 2a. From the PXRD patterns, the COF structures in the composite were still intact, and the key diffraction peak (main peak at 11.55° corresponding to the (110) Bragg peak) for the COF pores nicely matched with that of the original COF, indicating a maintained crystalline structure after combination with the hydrophilic part. In contrast to the unstable PEG-CCM upon thermal treatment (>200 °C) by thermogravimetric analysis (TGA) (Fig. 3b), APTES-COF-1 possesses high thermal stability up to ~300 °C. PEG-CCM@APTES-COF-1 displayed weight loss below 200 °C, which was largely due to the removal of absorbed water^38^. PEG-CCM@APTES-COF-1 started decomposition at 200 °C, and the overall weight loss was 70.6% at 700 °C, confirming the presence of the main PEG-CCM fragment in the composite. The photoluminescence (PL) spectrum (Fig. 3c) of PEG-CCM@APTES-COF-1 exhibited a peak centered at 475 nm, which was blue shifted by 75 nm relative to that of free CCM, likely stemming from the interaction between the PEG-CCM polymers and APTES-COF-1. This interaction was further confirmed by ultraviolet–visible (UV–vis) adsorption analyses (Fig. 3d). No characteristic absorption band was observed for APTES-COF-1, while PEG-CCM@APTES-COF-1 had a strong absorption band centered at 350 nm, which was blue shifted by 70 nm relative to that of free CCM. These results suggested that CCM was inserted into the APTES-COF-1 pore and that PEG was present. The entire structure can be regarded as a micelle (APTES-COF-1 as the oil phase, and PEG-CCM as the surfactant).

The apparent surface area calculated from the Brunauer–Emmett–Teller (BET) model was 442 m^2^ g^−1^ for APTES-COF-1 (Fig. 3e), which was relatively low as compared to the activated COF-1 due to the extra weight of APTES in the framework. This is consistent with a smaller size of pores (1.53–1.12 nm; the inset shows the DFT pore size distribution) and their decreased volume for PEG_2000_-CCM@APTES-COF-1 (inset in Fig. 3e). The FT-IR spectra of APTES-COF-1 and PEG-CCM@APTES-COF-1 (Supplementary Fig. 11) showed strong peaks at 1378 and 1342 cm^−1^ arising from B–O stretching vibrations present in APTES-COF-1. Notably, the FT-IR spectrum of PEG-CCM@APTES-COF-1 showed a distinct peak at 1572 cm^−1^ assigned to the carbonyl unit in PEG-CCM, which further confirmed the encapsulation of CCM in APTES-COF-1. Based on the foregoing results, PEG-CCM molecules were successfully grafted onto the APTES-COF-1 surface. Furthermore, PEG_2000_-CCM@APTES-COF-1 displayed the highest fluorescence intensity (Fig. 3f), clearly indicating that the introduction of PEG-CCM could dramatically alter the dispersibility of APTES-COF-1 and solubility of CCM in aqueous media. In addition, the zeta potential of PEG-CCM@APTES-COF-1 was ca. +3.4 mV, which was distinctively different from that of APTES-COF-1 (+1.3 mV), further suggesting that CCM was inserted into the APTES-COF-1 pores and that PEG was present.

### In vitro cellular uptake and antitumor efficacy

After full characterization of the PEG-CCM@APTES-COF-1 nanocomposites, we proceeded to explore their potential as drug-delivery carriers for therapeutic applications. To this end, we developed a facile and simple protocol for the encapsulation of drugs inside COFs via a hydrophobic effect, instead of using a complicated and tedious covalent modification approach. To the best of our knowledge, this represents the first attempt to incorporate the anticancer drug DOX inside COFs. Upon the encapsulation of DOX into APTES-COF-1, the zeta potential of APTES-COF-1@DOX dramatically changed from a positive state into a negative state. Evidently, APTES-COF-1 exhibited an extremely low loading of hydrophobic DOX molecules owing to its hydrophobic nature. However, after self-assembly with PEG-CCM, the PEG-CCM@APTES-COF-1 nanocomposites exhibited a boosted loading of DOX, for example, PEG_2000_-CCM@APTES-COF-1 had a considerably enhanced drug loading capability of 9.71 ± 0.13 wt% and high encapsulation efficiency of 90.5 ± 4.1% compared to bare APTES-COF-1. Notably, this loading efficiency is among the highest value for existing drug-loaded COF systems^22,23^ and comparable to the best MOF carrier system^26,28,39^.

**Fig. 4:**
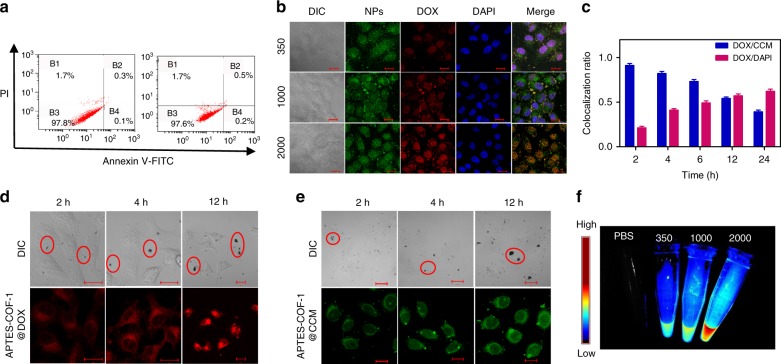
In vitro cytotoxicity and cellular uptake. **a** Flow cytometry analysis of HeLa cells incubated with blank (left) and APTES-COF-1 (right). The four areas represent the different phases of the cells: necrotic (B1), late-stage apoptotic (B2), early apoptotic (B4), and live (B3). **b** Confocal images of HeLa cells recorded at 6 h after co-incubation with the three different DOX (red channel)-loaded PEG-CCM@APTES-COF-1 nanoparticles (NPs) (green channel). **c** Co-localization ratio analysis on HeLa cells between the red channel from DOX and green channel from CCM and that between the red channel from DOX and the blue channel from the stained nuclei. Each data point represents the average and SD (one above and one below for the error bars), as measured from five different analysis in a single experiment. **d** Confocal images of HeLa cells after treatment with DOX-loaded APTES-COF-1 at different times corresponding to in vivo release. The inset shows the corresponding large APTES-COF-1 aggregates of the area within the dotted circle on the cell surface. **e** Confocal images of HeLa cells after treatment with CCM-loaded APTES-COF-1 at different times corresponding to in vivo release. The inset shows the corresponding large APTES-COF-1 aggregates of the area on the cell surface. The blue channel was excited at 405 nm and collected between 450 and 500 nm; the green channel was excited at 488 nm and collected between 510 and 550 nm; the red channel was excited at 543 nm and collected between 590 and 650 nm. The scale bars are 20 μm. **f** Luminescent image of the CCM after addition of sample to the medium

Flow cytometry assay revealed that the APTES-COF-1 without DOX was nontoxic when the concentration was below 200 μg mL^−1^. After 24 h of incubation, over 95% of cells survived (Fig. 4a). We then explored the intracellular drug-delivery capability of PEG-CCM@APTES-COF-1 composites by loading the well-known anticancer drug DOX into the composites and evaluated their therapeutic performance in HeLa cell viability. Figure 4b showed the red emission of DOX overlapped well with the green emission of the CCM vector after 6 h of incubation. The apparent red emission in the nuclei and cytosols suggested that DOX was released from the three carriers. Further extending the incubation time to 12 h, the DOX payload subsequently entered the nuclei. Finally, the green emission from CCM spread throughout the whole cells (Supplementary Fig. 12), signifying an efficient internalization of the PEG-CCM@APTES-COF-1 carriers. In the meantime, the co-localization ratio calculated from the red channel fluorescence of DOX and the green channel fluorescence of CCM in CLSM images steadily decreased after 6 h of incubation (Fig. 4c). Simultaneously, the co-localization ratio of both DOX and 4′,6-diamidino-2-phenylindole (DAPI) remained high after 6 h of incubation. For the DOX-loaded APTES-COF-1 carriers, the co-localization ratio between the red channel of DOX and the blue channel fluorescence of stained DAPI persisted at 30% after 12 h of incubation (Supplementary Fig. 13). However, from the image of the APTES-COF-1@DOX (Fig. 4d) and APTES-COF-1@CCM systems (Fig. 4e), we observed the DOX or CCM signal inside the cells after 2 h of incubation. With the increment of incubation time, the fluorescent signal of the nucleus did not considerably increase. This result indicated that the presence of large APTES-COF-1 aggregates on the cell surface affects cell endocytosis, leading to weak intracellular fluorescence. Therefore, the water-stable PEG-CCM@APTES-COF-1 carriers can be facilely tuned by not only the hydrophilic chains from PEG but also the specific noncovalent interactions within the self-assembly, which may be advantageous for extending drug release and endocytosis. As a control (Supplementary Fig. 14a), upon extending the incubation time to 12 h, for the DOX-loaded PEG-CCM carriers (Supplementary Fig. 14b), the fluorescence density decreased in the nucleus, and the drug release decreased as well. But for the PEG-CCM@APTES-COF-1 vector (Supplementary Fig. 14c), the drug release still increased and the fluorescence enhancement showed that the contents of the storage drugs of the carrier were significantly different. The confocal images in Supplementary Fig. 14 indicate that the obtained particles are single species instead of a mixture of PEG-CCM@APTES-COF-1 and PEG-CCM micelles.

In vitro cytotoxicity was evaluated by the 3-[4,5-dimethylthialzol-2-yl]-2,5-diphenyltetrazolium bromide (MTT) assay to demonstrate the superiority of DOX release and cellular uptake in our system. Surprisingly, the DOX-loaded PEG-CCM@APTES-COF-1 remarkably inhibited HeLa cell growth even with a very low DOX concentration range (0–0.25 µg mL^−1^), whereas the control groups (i.e., APTES-COF-1 and free DOX) did not show considerable cell growth inhibition under the identical concentration range (Supplementary Fig. 15). These results confirmed that the DOX-loaded PEG-CCM@APTES-COF-1 was an efficient drug delivery platform that could effectively inhibit cancer cell growth at low dosage in chemotherapy, which could mainly be attributed to the positive surface potential beneficiary for cell internalization^40^ and the sustained DOX release in the cytoplasm. Moreover, the dynamic PEGylated DOX-loaded system is prone to supply more nanocarriers with higher surface PEG densities for accumulation in tumor cells in vivo, leading to advanced antitumor activities^41,42^. Notably, PEG_2000_-CCM@APTES-COF-1@DOX showed considerably enhanced cancer cell inhibition compared to APTES-COF-1@DOX, suggesting its vast potential to selectively kill more acidic cancerous cells than normal tissue/cells in vivo. Taken together, grafting a pharmacokinetic modifier (PEG) onto the APTES-COF-1 surface through self-assembly enhanced the biocompatibility of the composites and extended their blood circulation time, which was due to the reduced protein adsorption or circumventing uptake by the reticuloendothelial system^43,44^. The higher permeability and longer retention (EPR) of the carrier could promote the accumulation of drugs in tumors^45^. Therefore, PEG_2000_-CCM@APTES-COF-1@DOX could achieve more accumulation in tumor tissues.

### In vivo antitumor activity

**Fig. 5:**
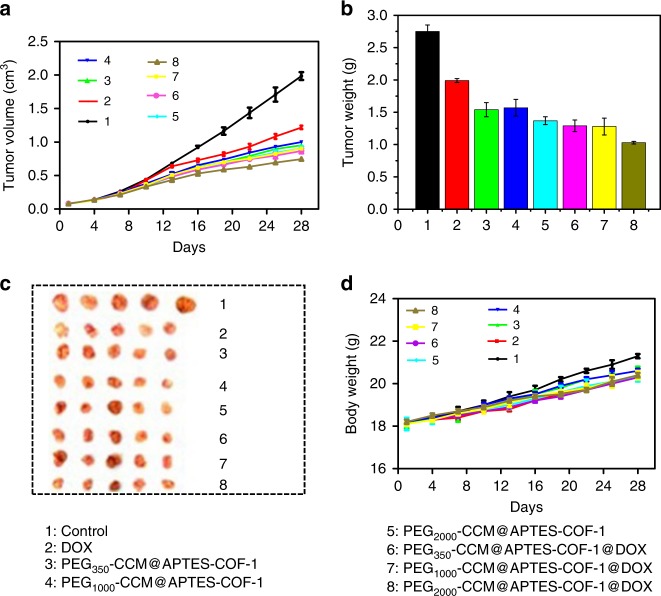
In vivo evaluation of antitumor efficacy. **a** Mean tumor volumes and **b** mean tumor weights after excision at day 28 (*n* = 5). **c** Representative photographs of mice tumors after treatment and **d** body weights of the mice in different groups after treatment at different time intervals. Relative antitumor assay after intravenous injection into the HeLa tumor-bearing mice with saline, free DOX, PEG_350_-CCM@APTES-COF-1, PEG_1000_-CCM@APTES-COF-1, PEG_2000_-CCM@APTES-COF-1, PEG_350_-CCM@APTES-COF-1@DOX, PEG_1000_-CCM@APTES-COF-1@DOX, and PEG_2000_-CCM@APTES-COF-1@DOX at an equivalent dose of 1.5 mg DOX kg^−1^ in PBS every 3 days. Data were presented as the mean ± SD (*n* = 5). Each data point represents the average and SD (one above and one below for the error bars), as measured from five different analysis in a single experiment

**Fig. 6:**
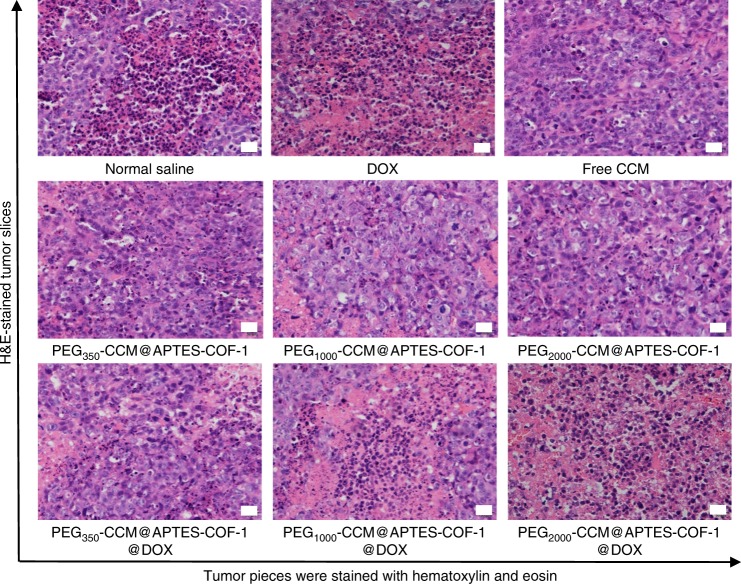
Immunohistochemical analysis. H&E-stained tumor slices collected from different groups of mice on the following day after various treatments. For all images: scale bar represents 50 µm

Inspired by the good biocompatibility and in vitro anticancer performance of PEG-CCM@APTES-COF-1@DOX, we investigated the in vivo drug-delivery and antitumor efficacy of the PEG-CCM@APTES-COF-1 nanocomposites in nude mice with a HeLa cell xenograft tumor model^46,47^. PEG-CCM@APTES-COF-1 with and without DOX were intravenously injected every 3 days for 28 days. In fact, Fig. 4f showed a quantitatively similar optical signal for unlabeled and PEG-CCM labeled APTES-COF-1 at the same concentration (3 mg mL^−1^), confirming that there was obvious fluorescence, in vivo fluorescence imaging. As indicated in Fig. 5a and Supplementary Fig. 16, sharp tumor growth suppression in mice was observed without noticeable relapse in the PEG-CCM@APTES-COF-1 group. Meanwhile, the weights and sizes of tumors isolated 28 days after treatment (Fig. 5b, c) were in accordance with the volumes, and no obvious difference in weight was discovered in each group of mice (Fig. 5d), implying that the PEG-CCM@APTES-COF-1 nanocomposites did not exert any side effects during the treatment. To further probe the underlying mechanism for the effective antitumor efficacy, representative hematoxylin and eosin (H&E) staining and immunohistochemical analysis were performed (Fig. 6). Histology analysis further revealed that considerable large-area apoptotic or necrotic regions were observed in the tumor tissues from the PEG-CCM@APTES-COF-1@DOX group composites, whereas no apparent inflammation or lesions were observed in the morphology of the normal organs. Consistent with the aforementioned in vitro findings, PEG_2000_-CCM@APTES-COF-1@DOX exhibited the highest antitumor efficiency, primary due to the synergy of the tumor-site-specific cellular uptake and DOX release. As PEG_2000_-CCM@APTES-COF-1@DOX circulates in the bloodstream (where the pH = ~7.4 in physiological conditions), DOX molecules could be retained in PEG2000-CCM@APTES-COF-1. After PEG_2000_-CCM@APTES-COF-1@DOX enters tumor cell, DOX molecules could be gradually released.

**Fig. 7:**
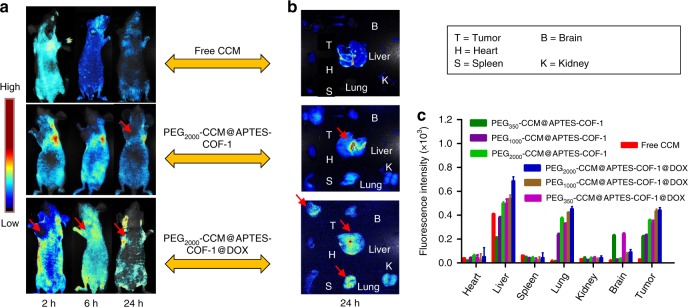
Fluorescence imaging and fluorescence intensity characterizations. **a** Ex vivo fluorescence images (ex: 455 nm) and **b** in vitro fluorescence images of major organs and tumors of mice after intravenous injection of PEG_2000_-CCM@APTES-COF-1, PEG_2000_-CCM@APTES-COF-1@DOX over a period of 24 h. **c** Mean CCM fluorescence intensities of major organs and tumor at 24 h after administration. Tail injection into the HeLa tumor-bearing mice was at a single sample dose of 5 mg kg^−1^ in PBS (3 mice/group). Each data point represents the average and SD (one above and one below for the error bars) as measured from five different analysis in a single experiment

In addition, the bio-distribution of each sample was tested by direct tracking the intrinsic fluorescence of CCM in organs ex vivo after intravenous injection of the samples into tumor-bearing mice through a tail vein. As shown in Fig. 7a, the fluorescence properties of CCM were employed to monitor the in vivo distribution of a series of samples in HeLa tumor-bearing mice at 2, 4, and 24 h. As indicated by the imaging, the DOX-loaded PEG-CCM@APTES-COF-1 group mice presented strong fluorescence from the tumor in comparison to the control. The tumor and various organs were further collected from the DOX-loaded system mice and analyzed by fluorescence imaging (Fig. 7b). Most of the DOX-loaded PEG-CCM@APTES-COF-1 accumulated in the liver, lung, and tumor tissue via intravenous injection, while minor distributions were distributed in the kidney, brain, spleen, and heart. However, the fluorescence signals of PEG_2000_-CCM@APTES-COF-1 and PEG_2000_-CCM@APTES-COF-1@DOX were higher in the tumor tissue than the other organs after 24 h of injection. PEG_2000_-CCM@APTES-COF-1@DOX exhibited the highest tumor accumulation. For example, the fluorescence intensity of PEG_2000_-CCM@APTES-COF-1@DOX in tumor tissue was 1.7-, 1.8-, 1.6-, and 8.3-fold higher than those of PEG_350_-CCM@APTES-COF-1, PEG_1000_-CCM@APTES-COF-1, PEG_2000_-CCM@APTES-COF-1, and free DOX after 24 h, respectively. The accumulation of DOX-loaded system mice intravenously injected in the liver and lung may be related to the reticuloendothelial system of the liver and lung, which is consistent with the previous results^47–49^. As expected, in comparison to the free CCM-treated mice, the mice treated with PEG-CCM@APTES-COF-1@DOX showed a considerably enhanced fluorescence intensity signal of CCM at the tumor site within the monitored time period (Fig. 7c). All the results convincingly demonstrated that the PEG-CCM@APTES-COF-1@DOX system, particularly with dynamic PEGylation 2000, can effectively reach tumor sites via an EPR effect and exhibit prolonged circulation in the bloodstream, resulting in the high antitumor efficacy of the DOX-loaded APTES-COF-1 nanocomposite system.

**Fig. 8:**
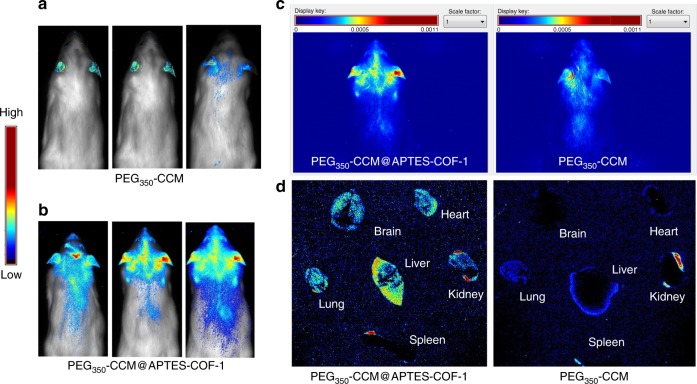
In vivo fluorescence imaging for brain tumor targeting and biodistribution. **a, b** In vivo fluorescence imaging of brains collected from ICR mice treated with PEG_350_-CCM@APTES-COF-1 (up) or PEG_350_-CCM (down) at 4 h post injection. **c** In vivo fluorescence imaging for brain tumor targeting and biodistribution under the same conditions. **d** Ex vivo fluorescence imaging of organs from ICR mice treated with PEG_350_-CCM@APTES-COF-1 or PEG_350_-CCM. The organs from top to bottom are as follows: brain, heart, liver, lung, kidney, and spleen

Taken together, our study showcases a facile synthesis of water-dispersible PEG-CCM@APTES-COF-1 nanocomposites as smart carriers for drug delivery with remarkable anti-cancer therapeutic efficiency. As far as we know, this represents the first study pertaining to a facile methodology to prepare monofunctional PEG-CCM@APTES-COF drug-delivery nanocarriers for therapeutic applications. Even more intriguingly, at 4 h post injection, the mouse (ICR mice and BALB/C nude mice) brains and organs were collected for in vivo (Fig. 8a, b and Supplementary Fig. 17a,c) and ex vivo imagings (Fig. 8d and Supplementary Fig. 17b,d). The fluorescence intensity of the PEG_350_-CCM@APTES-COF-1 at the brain tumor site was significantly higher than that for unassembled PEG_350_-CCM under the same conditions. (Fig. 8c). This result clearly showed that the PEG_350_-CCM@APTES-COF-1 nanoassemblies accumulated in the brain. In this study, the specific role of PEG_350_-CCM encapsulated inside the APTES-COF-1 in the brain targeting test had been elucidated, and further investigation is currently underway.

## Discussion

In conclusion, we have demonstrated a simple and facile one-pot process to afford water-dispersible PEG-CCM@APTES-COF nanocomposites as carriers for in vivo drug delivery and anticancer therapeutic applications. The surface modification of APTES-COF-1 with a pharmacokinetic modifier, PEG-CCM, considerably enhanced the biocompatibility of the nanocomposites and prolonged their blood circulation time. Moreover, we demonstrated that the nanocomposites were endocytosed by cells, and the pH-triggered disintegration of APTES-COF-1 in these acidic compartments led to efficient drug release. PEG-CCM@APTES-COF-1@DOX displayed higher penetration, longer retention, and a better tumor-inhibiting effect than the APTES-COF-1@DOX system. Drug distribution and ex vivo fluorescence imaging revealed that PEG_2000_-CCM@APTES-COF-1@DOX accumulated more drug in tumor tissues than other groups. In vivo antitumor examination further demonstrated that PEG_2000_-CCM@APTES-COF-1@DOX possessed superior tumor-inhibiting effect in tumor-bearing mice compared with those of the free DOX and other PEG-CCM@APTES-COF-1@DOX groups.

By offering the first insight into the endocytosis and in vivo antitumor activity of COFs, we anticipate that such drug-delivery systems will facilitate innovations and applications in cancer diagnostics and treatment. Besides, COFs modified with polymers also have potential applications in brain tumor imaging and therapy.

## Methods

### Chemicals and reagents

Curcumin, 2-propynylamine, 4-dimethylaminopyridine (DMAP), MTT, and *N*-(3-dimethylaminopropyl)-*N*′-ethylcarbodiimide hydrochloride were acquired from Sigma-Aldrich Inc. (St. Louis, MO). Doxorubicin hydrochloride (DOX•HCl) was purchased from Iffect Chemphar Co., Ltd. Ultrapure water with a resistivity of 18.2 MΩ cm^−1^ was used in all experiments. Bifunctional PEG polymers (PEG-OH, MW = 350, 1000, 2000 Da) were purchased from Shanghai Peng Shuo Biological Technology Co., Ltd. (Shanghai, China). Triethylamine (TEA) and dichloromethane (CH_2_Cl_2_) were predried over CaH_2_ and distilled prior to use. Tetrahydrofuran (THF) was predried over sodium shaving and distilled prior to use. Annexin V-FITC apoptosis detection kit was purchased from BD Biosciences (USA). All other reagents were purchased from Sinopharm Chemical Reagent Co., Ltd. and used as received without further purification. We obtained Hela cells from Institute of Biochemistry and Cell Biology, Shanghai Institute for Biological Science of Chinese Academy of Science (Shanghai, China). DEME/high glucose medium supplemented with 10% fetal bovine serum and 1% antibiotics (penicillin/streptomycin) were used to seed Hela cells. The cultures were maintained at 37 °C under a humidified atmosphere containing 5% CO_2_. Animal studies were performed under approved guidelines and also reviewed by the Institutional Animal Care and Use Committee of Simcere Laboratory Animal Center. Adult Sprague−Dawley rats (200 ± 20 g) and BALB/C nude mice aged 6 weeks were provided by Shanghai Ling Cheong Biotechnology Co., Ltd. The tumor volume was calculated using the reported method^50^. The tumor volume was defined as follows: *V* = *W*^2^*L*/2, in which *W* was the shortest diameter and *L* was the longest diameter. The tumor model was established by hypodermic injection of 1 × 10^7^ HeLa cells at the selected positions of BALB/C nude mice.

### Instrumentation

Transmission electron microscopy (TEM) and SAED images were acquired on a JEM-2100 electron microscope (JEOL, Japan) at an accelerating voltage of 100–120 kV. TEM samples were prepared by air-drying a drop of the nanocarriers solution on the surface of an ultrathin carbon film supported on copper grids. DLS measurements were performed on a BI-200SM (Brookhaven Instruments Corporation) with a He–Ne laser (*λ* = 633 nm). DLS samples were prepared by dissolving particles in THF or water and filtered through 0.22-μm filters. Laser scanning confocal images were recorded on LSM-710 (Zeiss Inc., Germany) and Leica (Leica TCS SP8 STED 3X, Germany) microscopes. MALDI-TOF MS measurements were performed on an autoflex II TOF/TOF mass spectrometer (Bruker Daltonics, Bremen, Germany) using 2,5-dihydroxybenzoic acid as the matrix. PXRD patterns were acquired using a D8 Advance X-ray diffractometer (Bruker) with a Cu *K*α radiation source (40 kV, 40 mA, *λ* = 1.54051 Å). NMR spectra were recorded on a Bruker AM-400 spectrometer with CDCl_3_ as the solvent. UV–vis spectra were obtained on a UV-1800(PC) UV–vis spectrophotometer (Mapada, China). TGA from 20 to 800 °C at 10 °C min^−1^ ramping was carried out on a PerkinElmer TG: DTA6300 under a nitrogen atmosphere. Fourier transform infrared (FT-IR) spectra were measured using a Vector-22 spectrometer (Bruker). ESI-MS was performed using a Finnigan LCQ mass spectrometer (ThermoFinnigan, San Jose, CA). Fluorescence spectra were recorded using a FluoroMax-4 spectrofluorometer (HORIBA Scientific, Japan) with an excitation and emission slit width of 5 nm. A Coulter FC-500 flow cytometer (Beckman-Coulter) was used for the flow cytometric analysis.

### Synthesis of APTES-COF-1

A tube was charged with 1,4-benzenediboronic acid (0.165 g, 1 mol), 0.05 g of APTES, 2.5 mL of 1,4-dioxane, and 2.5 mL of mesitylene in a reaction tube equipped with a magnetic stirring bar^21,25^. The tube was carefully degassed by three freeze–pump–thaw cycles and then sealed under vacuum. The reaction mixture was sonicated for 60 min. After being thermostated at 75 °C in an oil bath and stirred for 24 h, the reaction tube was cooled and opened; the mixture was then precipitated into an excess of 1,4-dioxane. COF-1 was obtained as a pure white powder (0.117 g, 70.9% yield).

### Synthesis of CCM-COOH

Curcumin (2.01 g, 5.46 mmol), DMAP (112 mg, 0.92 mmol), and Et_3_N (1.33 mL, 9.55 mmol) were dissolved in anhydrous THF (100 mL)^37^. Afterward, succinic anhydride (0.601 g, 6 mmol, 95%) in 5 mL of THF was added dropwise to the curcumin solution, followed by stirring and refluxing the mixture under argon overnight. The solvent was removed under reduced pressure, followed by adding EtOAc (55 mL) and 1 M HCl (15 mL). After stirring the mixture for another 10 min, the organic phase was separated and extracted with EtOAc three times; the solvent was removed under reduced pressure. The crude product was further purified by column chromatography (Yield: 69 %) with CH_2_Cl_2_/MeOH (95:5).

### Synthesis of PEG-CCM system

EDCI (383 mg, 2.0 mmol), DMAP (19 mg, 0.16 mmol), and CCM-COOH (404.5 mg, 0.88 mmol) were added to a solution of PEG_350_ (280 mg, 0.80 mmol) in a mixture of anhydrous THF/CH_2_Cl_2_ (1:1, 10 mL) at 0 °C. The reaction mixture was stirred for 4 h. Subsequently, the reaction mixture was diluted with CH_2_Cl_2_ (15 mL) and H_2_O (15 mL). The organic layer was separated, dried over MgSO_4_, and the solvent was removed under rotary evaporation. The mixture was then precipitated into an excess of diethyl ether. The above dissolution–precipitation cycle was repeated for three times. PEG_350_-CCM was obtained as a yellow powder (415.2 mg, 60.7%). Similar procedures were used in the synthesis of PEG_1000_-CCM (0.8 g, 0.80 mmol) and PEG_2000_-CCM (1.6 g, 0.80 mmol).

### Self-assembly of APTES-COF-1 and PEG-CCM

Two milligrams of APTES-COF-1 and 2 mg of PEG_350_-CCM were dispersed in 1 mL of 1,4-dioxane, stirred and maintained at a predetermined temperature with a water bath for 20 min. Afterwards, 2 mL of water was slowly added within 1 h, followed by the injection of 7 mL of water within 1 h. After stirring for another 2 h, residual PEG_350_-CCM and 1,4-dioxane were removed by dialysis (MWCO 1 kDa) against deionized water, after freeze-drying. According to similar procedures, PEG_1000_-CCM@APTES-COF-1 and PEG_2000_-CCM@APTES-COF-1 were also synthesized by self-assembly.

### Self-assembly of APTES-COF-1@DOX and PEG-CCM

The encapsulation of hydrophilic molecules DOX was performed as follows. In total, 2 mg of APTES-COF-1 was dissolved in 1 mL of 1,4-dioxane, stirred and maintained at a predetermined temperature with a water bath for 20 min. In total, 16 mg of DOX was dissolved in of 2 mL of water and the aqueous solution was slowly added within 1 h, followed by the injection of 7 mL of water within 1 h. After stirring for another 2 h, 1, 4-dioxane and the free DOX were removed by dialysis (MWCO 1 kDa) against deionized water.

In a typical self-assembly experiment, 2 mg of APTES-COF-1@DOX and 2 mg of PEG_350_-CCM were dispersed in 1 mL of 1,4-dioxane, stirred and maintained at a predetermined temperature with a water bath for 20 min. Afterwards, 2 mL of water was slowly added within 1 h, followed by the injection of 7 mL of water within 1 h. After stirring for another 2 h, residual PEG_350_-CCM and 1,4-dioxane were removed by dialysis (MWCO 1 kDa) against deionized water, after freeze-drying. According to similar procedures, PEG_1000_-CCM@APTES-COF-1@DOX and PEG_2000_-CCM@APTES-COF-1@DOX were also synthesized by self-assembly.

### In vitro cellular uptake

The qualitative cellular uptake of the PEG-CCM@APTES-COF-1 and PEG-CCM@APTES-COF-1@DOX was observed by CLSM (LSM-710; Zeiss Inc., Germany) similar analysis methods^51^. The cells were seeded into 12-well plate with a density of 10^5^ cells in each well, and incubated with the medium for 24 h. Then, the culture medium was replaced by DMEM with different concentrations of nanocomposites. After 2 h, 6 h, and 12 h incubation, the medium in each well was replaced into PBS. The cell nucleus was stained with DAPI, and the fluorescence intensity was reported by confocal fluorescence microscopy at an excitation of 405 nm. The CCM and DOX signals were recorded at an excitation of 488 nm.

### Flow cytometry analysis

The quantitative cellular uptake of the PEG-CCM@APTES-COF-1 and PEG-CCM@APTES-COF-1@DOX was accomplished using flow cytometry similar analysis methods^52^. HeLa cells were seeded in a six-well plate for 12 h containing 2 mL of medium and washed with PBS and then harvested by 0.25% trypsin-EDTA. These cells were incubated with blank and APTES-COF-1 as described above for 24 h. The resulting cells were centrifuged at 1000 rpm for 5 min at 4 °C and stained with the mixture of 2.0 μL of Annexin V-FITC and 2.0 μL of propidium iodide (PI) for 15 min. After three cycles of washing and centrifugation, the cells were resuspended in PBS and analyzed with flow cytometry (Beckman Coulter, CA, USA) over FL1 (Annexin V-FITC) and FL3 (PI) channels.

### In vitro cell viability

The cell viability of the PEG-CCM@APTES-COF-1@DOX was assayed using a MTT assay reported previously^50^. HeLa cells were seeded at a density of 1 × 10^4^ cells per well in 96-well plates, preincubated for 24 h, and subsequently treated with PEG_350_-CCM@APTES-COF-1@DOX, PEG_1000_-CCM@APTES-COF-1@DOX, free DOX, and PEG_2000_-CCM@APTES-COF-1@DOX with different DOX concentrations. After incubation for 24 h, 20 μL of MTT solution (5 mg mL^−1^) in PBS (pH 7.4) was added to each well and the cells were incubated for another 2 h. Afterwards, the medium containing unreacted MTT was removed carefully, followed by adding 150 μL of DMSO to each well to dissolve the MTT-formazan crystals. Next, light absorbances (abs.) at *λ* = 490 nm were recorded in a TRITURUS microplate reader (Perkin Elmer Singapore Pte. Ltd. Singapore). The average value of four independent experiments was calculated and the relative cell viability was measured by the following equation: cell viability (%) = (mean of abs. value of treatment group/mean abs. value of control) × 100%.

### Immunohistochemistry

Tumors were fixed in 10% (w/v) neutral buffered formalin and the fixed tissues were embedded in paraffin from which continuous 4 μm sections were prepared. Hematoxylin and eosin (H&E) staining was carried out based on the standard protocol. For immunohistochemistry, heat-induced antigen retrieval was performed in sodium citrate buffer (pH 6.0). A rabbit polyclonal anti-HDAC1 antibody (Cat. ab7028, Abcam, USA) was used at a 1:100 dilutions. Briefly, cryogenic slides were prepared and fixed with 10% formalin for 30 min at room temperature. After washing with running DI water for 5 min, the slides were treated with gradient concentrations of alcohol (100, 95, and 70%), for 20 s each. The hematoxylin staining was performed for 3 min and washed with water for 1 min. The eosin staining was performed for 1 min. The slides were washed, treated with xylene, and mounted with Canada balsam. The images were acquired with an optical microscope.

### Animals and tumor models

Inspired by the high accumulation of PEG-CCM@APTES-COF-1@DOX at the tumor site and their in vitro self-assisted anticancer activity, we further investigated the in vivo anticancer effect on BALB/C mice with HeLa tumor. When tumors were increased to ∼150–200 mm^3^ in volume, the mice were randomly divided into eight groups, and each group involved five mice. HeLa tumor-bearing mice were treated with different nanocomposites by intravenous injection for 28 days at the dose of 5 mg kg^−1^ per mouse (3 day)^−1^. Each mouse was earmarked and followed individually throughout the whole experiment. The tumor volume and body weight were then determined according to the method as described above every 3 days for 4 weeks until the euthanasia. They were treated as follows: group 1, intravenous injection of 0.9% NaCl; group 2, intravenous injection of the free DOX injection; group 3, intravenous injection of the PEG_350_-CCM@APTES-COF-1; group 4, intravenous injection of the PEG_1000_-CCM@APTES-COF-1; group 5, intravenous injection of the PEG_2000_-CCM@APTES-COF-1; group 6, intravenous injection of the PEG_350_-CCM@APTES-COF-1@DOX; group 7, intravenous injection of the PEG_1000_-CCM@APTES-COF-1@DOX; group 8, intravenous injection of the PEG_2000_-CCM@APTES-COF-1@DOX. The therapeutic effects were evaluated by monitoring the changes of relative body weight and relative tumor volume of the mice in different groups. After 28 days of treatment, the mice were sacrificed and the major organs and tumors were collected for further analysis. Tumor growth was inhibited after the mice were intravenously injected with the free DOX and PEG-CCM@APTES-COF-1@DOX, compared with 0.9% NaCl and the PEG-CCM@APTES-COF-1 as a negative and positive control, respectively.

### Data availability

All relevant data supporting the findings of this study are available from the corresponding authors on request.

## Electronic supplementary material

Supplementary Information
