# Supplementary material for: Water-dispersible PEG-curcumin/amine-functionalized covalent organic framework nanocomposites as smart carriers for in vivo drug delivery
Source: Nat Commun. 2018 Jul 17;9:2785. doi: 10.1038/s41467-018-04910-5 (PMC6050241; doi:10.1038/s41467-018-04910-5)
Supplement: Supplementary file 1 — Supplementary Information [file 41467_2018_4910_MOESM1_ESM.docx]

**Supplementary Information**

**Supplementary Figure 1. Syntheses of a series of polymer@COFs.** (**a, b**) The synthesis of PEG-CCM amphiphilic block copolymers containing hydrophobic CCM blocks. (**c, d**) Schematic representation of APTES-COF-1 based nanocomposites self-assembled from three diﬀerent polyethylene glycol (PEG) modiﬁed monofunctional curcumin derivatives (PEG-CCM).

**Supplementary Figure 2**. The corresponding structural model. Encapsulation of DOX molecules into APTES-COF-1 occurs during the self-assembly of PEG-CCM@APTES-COF-1.


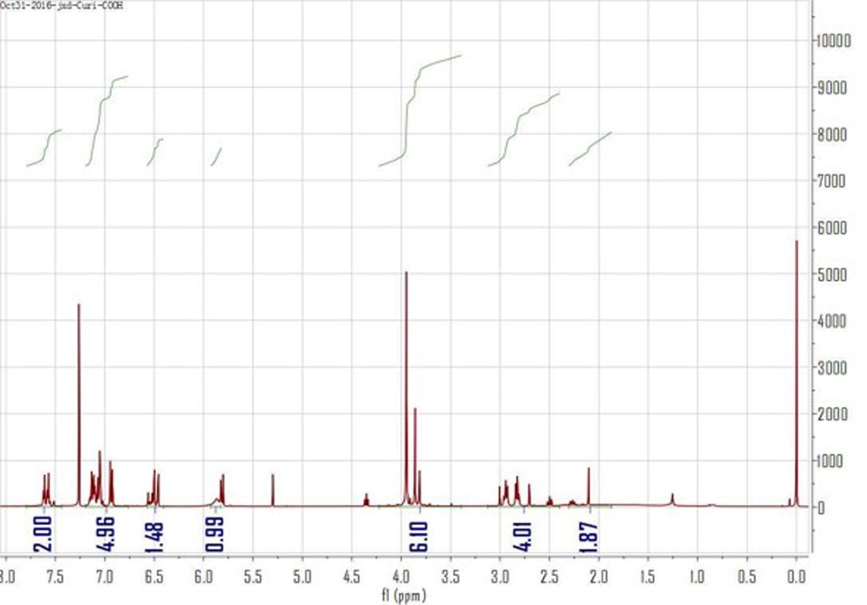


**Supplementary Figure 3.** ^1^H NMR spectra (CDCl_3_) of curcumin derivatives (CCM-COOH).


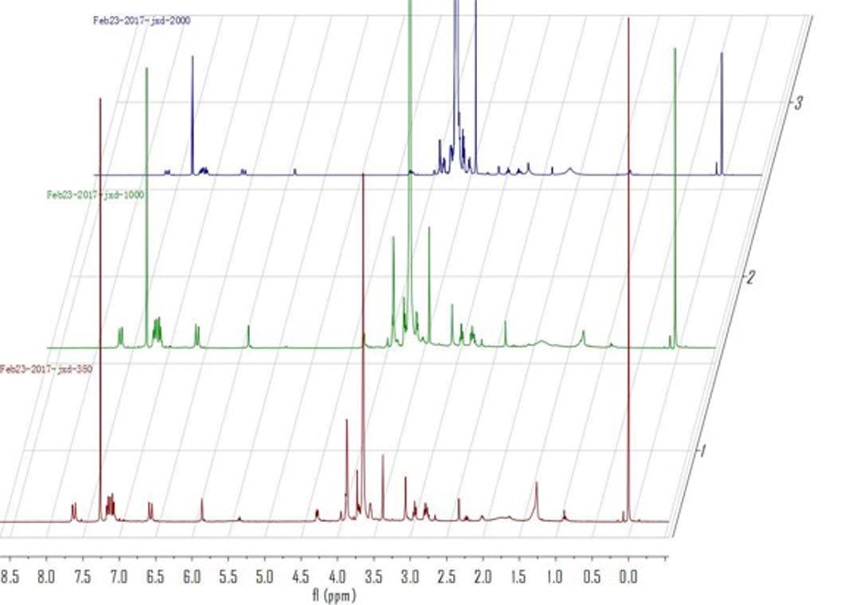


PEG_350_-CCM

PEG_1000_-CCM

PEG_2000_-CCM

**Supplementary Figure 4**. ^1^H NMR spectra of PEG_350_-CCM, PEG_1000_-CCM, and PEG_2000_-CCM monomer in CDCl_3_.


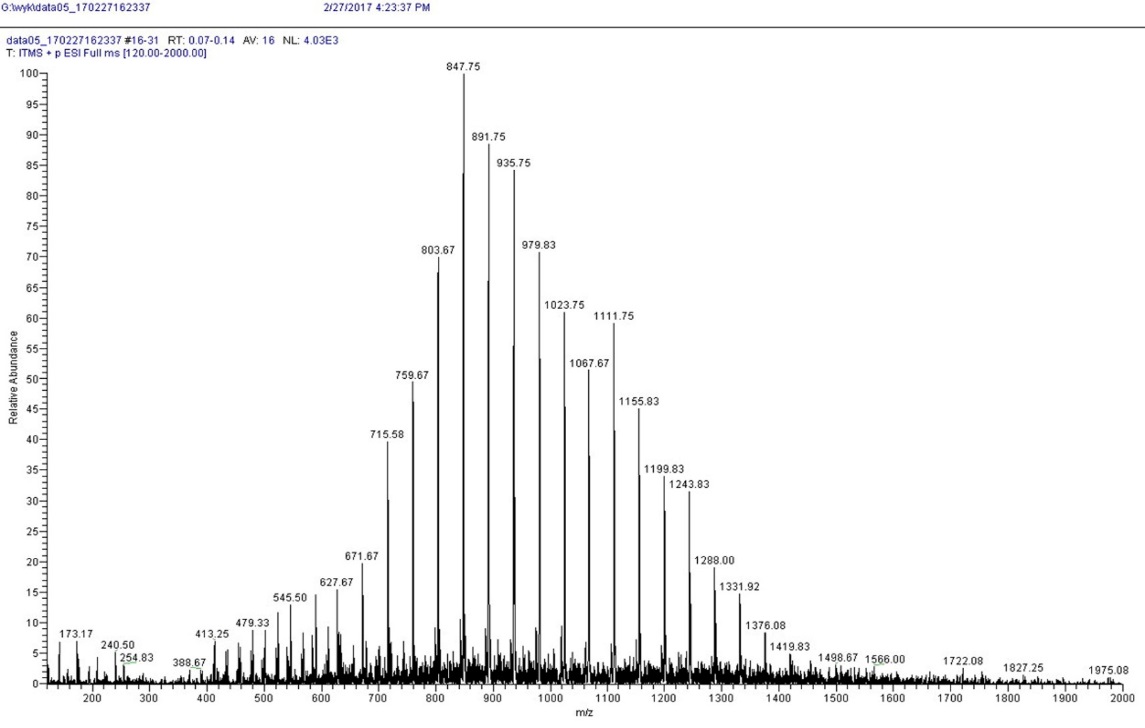


PEG_350_-CCM

**Supplementary Figure 5**. ESI-MS spectra of PEG_350_-CCM monomer.


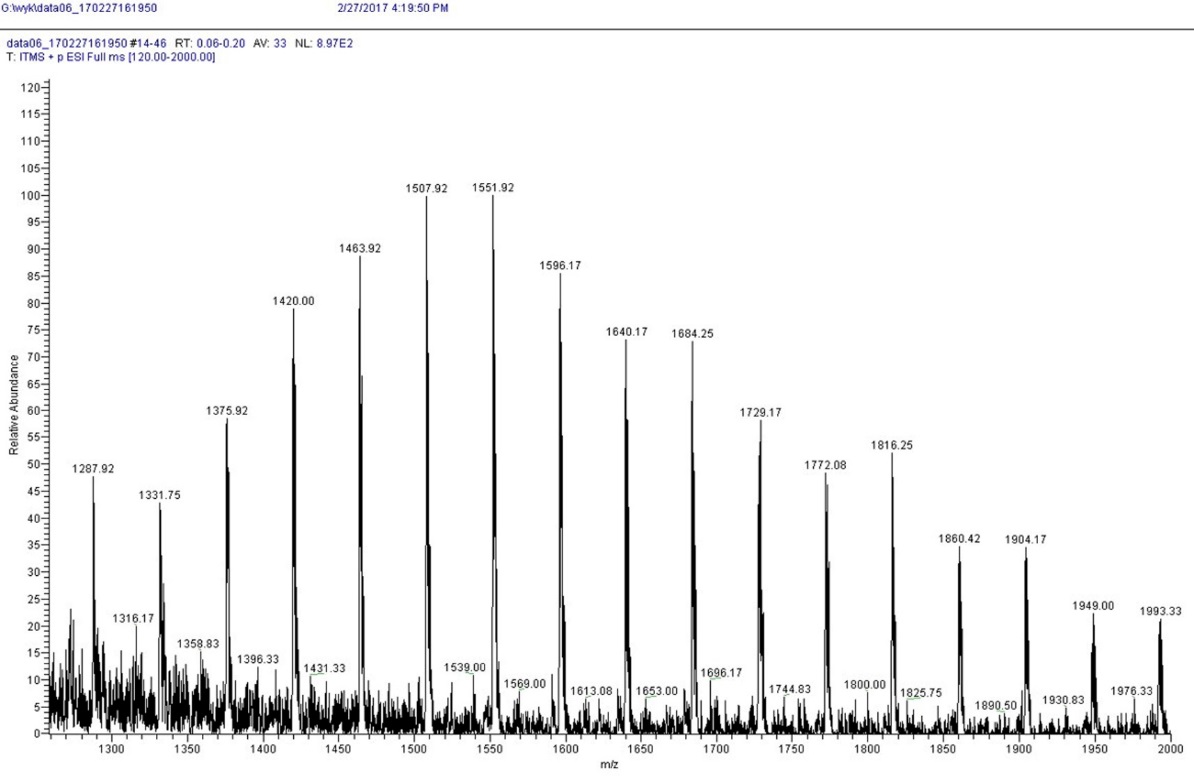


PEG_1000_-CCM

**Supplementary Figure 6.** ESI-MS spectra of PEG_1000_-CCM monomer.

**Supplementary Figure 7.** MALDI-TOF MS spectra of PEG_2000_-CCM.

**Supplementary Figure 8.** Particle size distributions of (**a**) APTES-COF-1, (**b**) PEG_350_-CCM@APTES-COF-1, (**c**) PEG_1000_-CCM@APTES-COF-1, and (**d**) PEG_2000_-CCM@APTES-COF-1 after 1 month determined by DLS, recorded in PBS buffer (pH 7.4, 10 mM) or DMSO/PBS mixture (v/v = 9/1) for (a) COF-1.

**Supplementary Figure 9**. TEM images of (**a**) PEG_350_-CCM, (**b**) PEG_1000_-CCM, and (**c**) PEG_2000_-CCM. For all images: scale bar: 0.5 µm.

**Supplementary Figure 10.** Particle size distributions of (**a**) PEG_350_-CCM, (**b**) PEG_1000_-CCM, and (**c**) PEG_2000_-CCM determined by DLS, recorded in PBS buffer (pH 7.4, 10 mM).


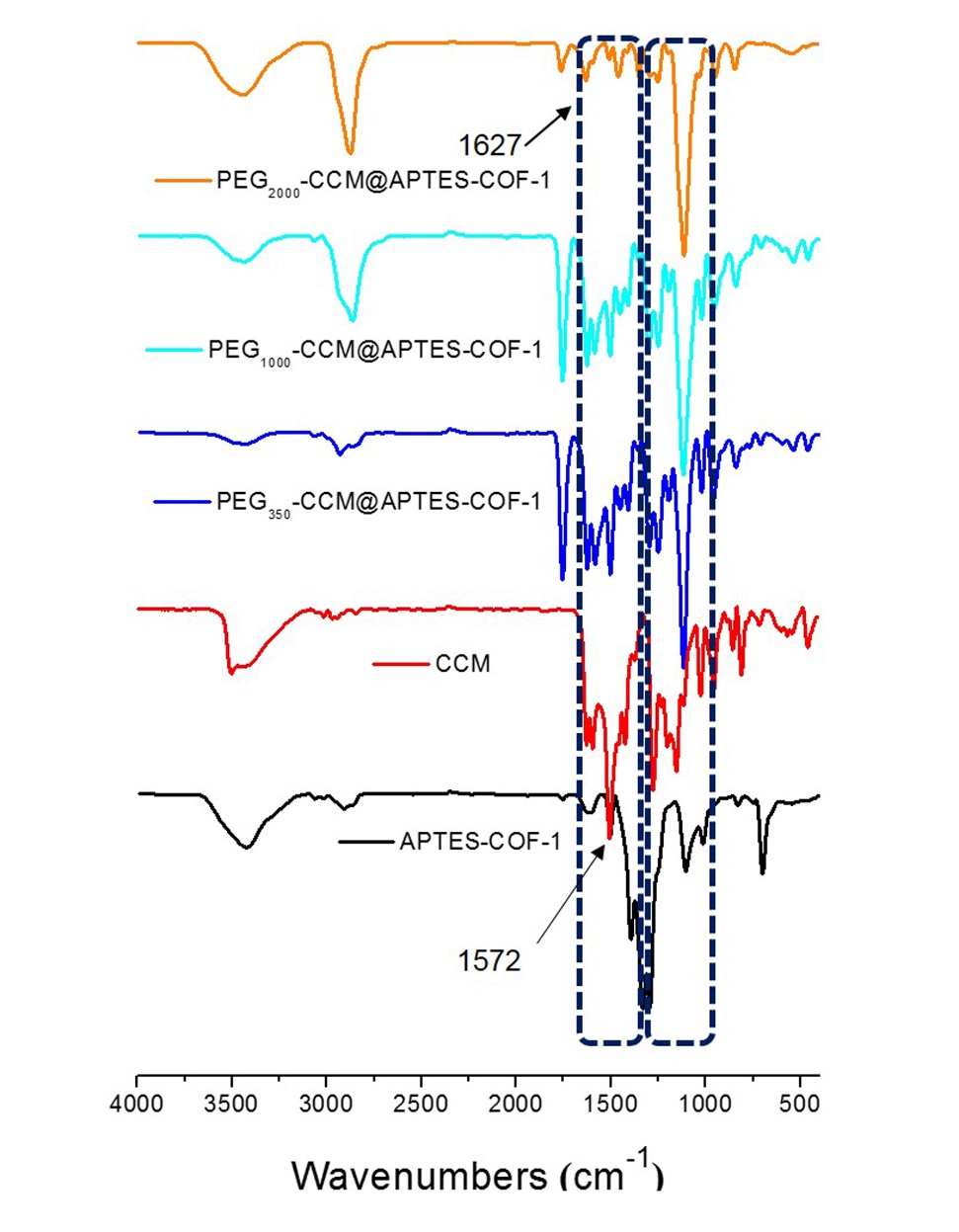


**Supplementary Figure 11.** FT-IR spectra of APTES-COF-1, CCM, PEG_350_-CCM@APTES-COF-1, PEG_1000_-CCM@APTES-COF-1, and PEG_2000_-CCM@APTES-COF-1.

**Supplementary Figure 12.** CLSM images of HeLa cells recorded three diﬀerent polyethylene glycol (PEG) modiﬁed monofunctional curcumin derivatives (PEG-CCM) after coincubation with DOX (red channel)-loaded PEG-CCM@APTES-COF-1 nanocomposites (green channel). The cell nuclei were stained with DAPI (blue channel). The blue channel was excited at 405 nm and collected between 450 and 500 nm; the green channel was excited at 488 nm and collected between 510 and 550 nm; the red channel was excited at 543 nm and collected between 590 and 650 nm. The scale bars are 20 μm.

**Supplementary Figure 13.** Co-localization ratio analysis between the red channel ﬂuorescence from DOX and the blue channel ﬂuorescence of stained DAPI.


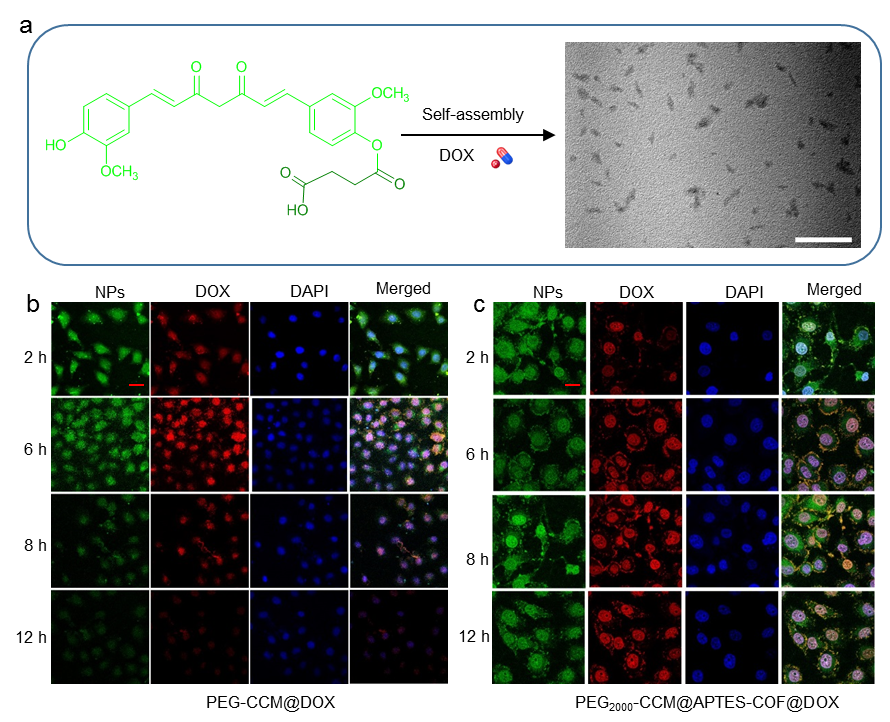


**Supplementary Figure 14.** **Polymer assembly and fluorescence imaging.** (**a**) Schematic representation for the synthesis of micelles *via* self-assembly. Scale bars, 500 nm. (**b**) Confocal images of HeLa cells recorded at the same time intervals after co-incubation with the DOX (red channel)-loaded PEG-CCM nanoparticles (NPs) (green channel). (**c**) Confocal images of HeLa cells after treatment with DOX-loaded PEG_2000_-CCM@APTES-COF-1 at different times corresponding to *in vivo* release. The yellow color represents the overlap between the NPs and DOX signals. The blue channel was excited at 405 nm and collected between 450 and 500 nm; the green channel was excited at 488 nm and collected between 510 and 550 nm; the red channel was excited at 543 nm and collected between 590 and 650 nm. The scale bars are 20 μm.


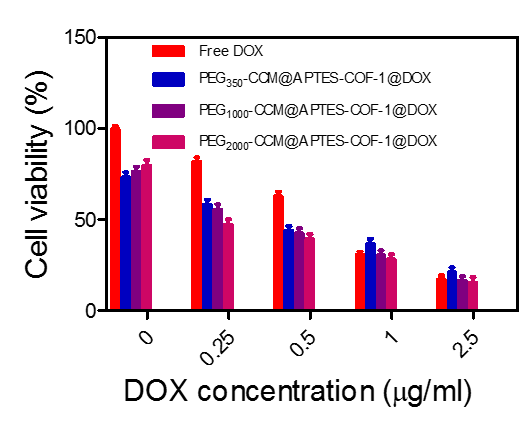


**Supplementary Figure 15.** *In vitro* viability of HeLa cells after 24 h of incubation with PEG_350_-CCM@APTES-COF-1@DOX, PEG_1000_-CCM@APTES-COF-1@DOX, PEG_2000_-CCM@APTES-COF-1@DOX and free DOX at different drug concentration.

**Supplementary Figure 16. Representative photographs of tumors collected from the mice after various treatments.** (**a**) PBS only, (**b**) DOX, (**c**) PEG_350_-CCM@APTES-COF-1, (**d**) PEG_1000_-CCM@APTES-COF-1, (**e**) PEG_2000_-CCM@APTES-COF-1, (**f**) PEG_350_-CCM@APTES-COF-1@DOX, (**g**) PEG_1000_-CCM@APTES-COF-1@DOX, and (**h**) PEG_2000_-CCM@APTES-COF-1@DOX. The monitoring cycle is 28 days. Every nanocomposites: 5mg/mL, 20 µL.

**Supplementary Figure 17.** (**a**, **c**) *In vivo* fluorescence imaging of brains collected from BALB/C nude mice treated with PEG_350_-CCM@APTES-COF-1 (up) or PEG_350_-CCM (down) at different times post-injection. (**b**, **d**) *Ex vivo* fluorescence imaging of organs from BALB/C nude mice treated with PEG_350_-CCM@APTES-COF-1 or PEG_350_-CCM. The organs from top to bottom are as follows: brain, heart, liver, lung, kidney and spleen.
